# Descripción de la micobiota de los tubos endotraqueales de pacientes de unidades de cuidados intensivos en Bogotá, Colombia

**DOI:** 10.7705/biomedica.6884

**Published:** 2023-08-31

**Authors:** Mónica Gabriela Huertas, Miguel Rodríguez, Patricia Castro, Sergio Danilo Cruz, Érika Alejandra Cifuentes, Andrés Felipe Yepes, María Mercedes Zambrano, Ana Margarita Baldión

**Affiliations:** 1 Genética Molecular, Corporación CorpoGen, Bogotá, D.C., Colombia Corporación CorpoGen Bogotá, D.C. Colombia; 2 Escuela de Medicina, Universidad Pedagógica y Tecnológica de Colombia, Tunja, Colombia Universidad Pedagógica y Tecnológica de Colombia Universidad Pedagógica y Tecnológica de Colombia Tunja Colombia; 3 Departamento de Patología y Laboratorios, Hospital Universitario Fundación Santa Fe de Bogotá, Bogotá, D.C., Colombia Hospital Universitario Fundación Santa Fe de Bogotá Bogotá, D.C. Colombia

**Keywords:** micobioma, microbiota, neumonía asociada con el respirador, intubación endotraqueal, unidades de cuidado intensivo, mycobiome, microbiota, pneumonia, ventilator-associated, intubation, intratracheal, intensive care units

## Abstract

**Introducción.:**

La colonización por microorganismos patógenos de los dispositivos médicos usados en las unidades de cuidados intensivos es un factor de riesgo para el aumento de infecciones asociadas con la atención en salud y, por lo tanto, al de la morbilidad y la mortalidad de los pacientes intubados. En Colombia, no se ha descrito la colonización por hongos de los tubos endotraqueales, con lo cual se podrían considerar nuevas opciones terapéuticas para el beneficio de los pacientes.

**Objetivo.:**

Describir los hongos que colonizan los tubos endotraqueales de los pacientes en unidades de cuidados intensivos, junto con su perfil de sensibilidad a los antifúngicos.

**Materiales y métodos.:**

Se realizó un estudio observacional, descriptivo, en dos centros hospitalarios durante 12 meses. Se recolectaron tubos endotraqueales de pacientes de las unidades de cuidados intensivos. Estos fueron procesados para cultivar e identificar hongos, y para establecer su perfil de sensibilidad a los antifúngicos.

**Resultados.:**

Se analizaron 121 tubos endotraqueales obtenidos de 113 pacientes. De estos, el 41,32 % se encontró colonizado por los hongos *Candida albicans* (64,61 %), *C*. no-*albicans* (30,77 %), *Cryptococcus* spp. (3,08 %) o mohos (1,54 %). Todos los hongos evaluados presentaron una gran sensibilidad a los antifúngicos, con un promedio del 91 %.

**Conclusión.:**

Se encontró colonización fúngica en los tubos endotraqueales de pacientes con asistencia respiratoria mecánica. El perfil de sensibilidad en estos pacientes fue favorable. Se requiere un estudio clínico para correlacionar los microorganismos colonizadores y su capacidad de generar infección.

## Abstract (en)

**Introduction.:**

Medical device colonization by pathogenic microorganisms is a risk factor for increasing infections associated with health care and, consequently, the morbidity and mortality of intubated patients. In Colombia, fungal colonization of endotracheal tubes has not been described, and this information could lead to new therapeutic options for the benefit of patients.

**Objective.:**

To describe the colonizing fungi of the endotracheal tubes from patients in the intensive care unit, along with its antifungal sensitivity profile.

**Materials and methods.:**

We conducted a descriptive, observational study in two health centers for 12 months. Endotracheal tubes were collected from patients in intensive care units. Samples were processed for culture, fungi identification, and antifungal sensitivity profile assessment.

**Results.:**

A total of 121 endotracheal tubes, obtained from 113 patients, were analyzed: 41.32 % of the tubes were colonized by *Candida albicans* (64.62%), *C*. non-*albicans* (30.77%), *Cryptococcus* spp. (3.08%) or molds (1.54%). All fungi evaluated showed a high sensitivity to antifungals, with a mean of 91%.

**Conclusion.:**

Fungal colonization was found in the endotracheal tubes of patients under invasive mechanical ventilation. The antifungal sensitivity profile in these patients was favorable. A clinical study is required to find possible correlations between the colonizing microorganisms and infectivity.

El uso de dispositivos médicos en las unidades de cuidados intensivos es un factor de riesgo para el aumento de las infecciones asociadas con la atención en salud. En Colombia, para el 2020, el 25,8 % de las infecciones asociadas con dispositivos tuvo como resultado clínico, la muerte. Se reportaron 1.666 casos, de los cuales el 31,8 % por neumonía asociada con el respirador ^1^. Los gérmenes más frecuentes fueron las bacterias gramnegativas (74 %), seguidas de las grampositivas (19 %), los hongos (4 %) y otros (2 %). *Pseudomonas aeruginosa* fue el patógeno más frecuente (20 %), seguido de *Staphylococcus aureus* (11 %) y *Klebsiella pneumoniae* (10 %). En la neumonía precoz, el agente patógeno más frecuente fue *S. aureus* y, en la tardía, *P. aeruginosa*^2^.

La neumonía asociada con el respirador se define como aquella que ocurre entre 48 y 72 horas después de la intubación y la asistencia respiratoria mecánica ^3^. Esta es la infección asociada con la atención de la salud más reportada en los pacientes en la unidad de cuidados intensivos y está asociada con mayor morbimortalidad e incremento de los costos ^4^. Es la segunda infección más frecuente asociada con dispositivos médicos, después de la asociada con catéteres, y es responsable del 30 % de las infecciones adquiridas en dicha unidad. Se estima que el 10 % de los pacientes que requieren asistencia respiratoria mecánica desarrollará una neumonía ^2^.

La intubación y la presencia del tubo endotraqueal se ha relacionado con un aumento entre tres y diez veces en el riesgo de padecer neumonía ^5^. La tasa de incidencia varía entre 4 y 50 casos por cada 100 pacientes ^6^, con una mortalidad de hasta el 76 % ^5^^,^^7^. Diversos estudios han demostrado que el tubo endotraqueal es colonizado por microbios orales pocas horas después de encontrarse en la vía aérea del paciente ^5^^,^^8^. Las neumonías ocurren entre el 8 y el 28 % de los pacientes intubados ^9^^-^^12^. Igualmente, se ha reconocido que la acumulación de microorganismos (bacterias y levaduras) en la luz del tubo endotraqueal puede generar biopelículas que, una vez terminan su ciclo, se desprenden y migran, y pueden introducirse en la vía aérea ^13^.

Las biopelículas son estructuras tridimensionales, biológicamente activas, conformadas por diversos microorganismos y pueden estar conformadas por una sola especie o por varias. Las biopelículas están embebidas en sustancias extracelulares producidas por los mismos microorganismos y tienen la capacidad de adherirse a distintos tipos de superficies bióticas y abióticas ^14^. Los microorganismos pueden unirse a superficies abióticas y, por lo tanto, convierten a los dispositivos médicos invasivos en un blanco importante de estudio y prevención de infecciones. Las muestras provenientes de secreciones traqueales indican que existe una relación entre los agentes causales de la neumonía asociada con el respirador y los microorganismos recuperados de biopelículas de tubos endotraqueales, en el 55 al 70 % de los casos ^15^.

La microbiota es el conjunto de microorganismos (bacterias, hongos, virus y parásitos) que viven en diferentes partes del cuerpo. Estas comunidades de microorganismos son dinámicas y cambian frente a diferentes estímulos o factores ^16^^,^^17^. Por otra parte, se deben diferenciar los sufijos -bioma (comunidad) y -oma (conjunto), y no confundirlos, como se hace en algunas ocasiones. El microbioma se refiere a todo el hábitat, con los microorganismos, sus genes y las condiciones ambientales ^17^. Los diferentes hongos que hacen parte del microbioma, forman lo que se conoce como micobiota ^18^^-^^20^.

El microbioma pulmonar parece jugar un papel relevante en la aparición de la neumonía asociada con el respirador; suele caracterizarse por una biomasa relativamente grande y el crecimiento excesivo de uno o más agentes patógenos ^21^. El número de estudios que describen el micobioma respiratorio es limitado. Sin embargo, en muestras de lavado broncoalveolar, se han reportado levaduras como *Saccharomyces cerevisiae*, *Candida* spp., *Meyerozyma guilliermondii* (*C. guilliermondii*), *Pichia jadini* y *Debaryomyces*; además, hongos filamentosos como *Cladosporium*, *Aspergillus* spp. y *Penicillium* spp. ^20^.

Es frecuente encontrar colonización de la vía aérea por hongos, en especial, por *Candida* spp. Aunque la neumonía por este agente es rara, puede causarla, así como también otras enfermedades invasivas en pacientes críticos. En estudios previos, se ha documentado hasta un 27 % de incidencia de colonización de tubos endotraqueales por especies fúngicas ^22^. La incidencia de invasión por infecciones fúngicas en cuidados intensivos está en aumento. En pacientes con neoplasias hematológicas o en pacientes sometidos a trasplante de órgano sólido o alogénico de progenitores hematopoyéticos, se ha observado una mortalidad entre el 20 y el 30 % a causa de aspergilosis pulmonar invasiva y, más del 80 %, cuando la infección es por cepas resistentes a los azoles ^23^.

Este estudio hizo parte de una investigación previa, cuyo objetivo era explorar y caracterizar la microbiota del tubo endotraqueal de pacientes colombianos. En dicho estudio, se observó que las bacterias de la microbiota de los pacientes intubados, incluían principalmente los filos *Proteobacteria*, *Firmicutes* y *Bacteroidetes*. Se correlacionó su presencia con la unidad de cuidados intensivos en la que los pacientes fueron hospitalizados, mientras que el tiempo de intubación y el diagnóstico de neumonía asociada con el respirador no mostraron una asociación significativa ^24^.

Teniendo en cuenta los reportes de colonización de los tubos endotraqueales por especies fúngicas y la posibilidad de que sean agentes causales de infecciones asociadas con la atención en salud, el presente análisis tuvo como objetivo describir los hongos colonizadores de los tubos endotraqueales de los pacientes de la unidad de cuidados intensivos y su perfil de resistencia a los antimicóticos.

## Materiales y métodos

### Descripción del estudio

Se llevó a cabo un estudio observacional, descriptivo, en el cual se recolectaron tubos endotraqueales de pacientes adultos, hospitalizados en las unidades de cuidados intensivos del Hospital Universitario Fundación Santa Fe de Bogotá y de la Clínica Colombia entre diciembre de 2016 y diciembre de 2017, con el objetivo de describir el micobioma de los tubos endotraqueales. El resultado clínico no es una variable de este estudio.

El muestreo se hizo por conveniencia e incluyó todos los tubos endotraqueales de los pacientes que requirieron asistencia respiratoria mecánica por un período de 48 horas o más. Se excluyeron muestras que no se habían procesado en las dos horas siguientes a su recolección. El protocolo de investigación fue aprobado por el comité de ética clínica de las instituciones participantes: el Hospital Universitario Fundación Santa Fe de Bogotá, Colombia (CCEI-5788-2016), y la Clínica Universitaria Colombia (CEIFUS 41617). Por el tipo de muestra, no fue necesario el consentimiento informado.

### Recolección de la muestra y cultivo de hongos

Las muestras fueron recolectadas siguiendo el protocolo establecido por el grupo de investigación, y fueron procesadas simultáneamente para el cultivo de bacterias y hongos. Los resultados de los análisis bacterianos hacen parte de otra publicación ^24^. Después de la extubación, realizada por el personal médico, los tubos endotraqueales se introdujeron en frascos estériles de 500 ml (Schott, Maguncia, Alemania), y se transportaron al Laboratorio de Microbiología de la Fundación Santa Fe de Bogotá. Las muestras se transportaron en una nevera de poliestireno, destinada únicamente para tal fin, a una temperatura de 4 °C, y se procesaron dentro de las dos horas siguientes a su recolección.

Se ejecutó un corte de 5 cm de la parte inferior distal del tubo, se suspendió en solución salina (0,85 % NaCl) y se sometió a tres ciclos de vórtex (30 segundos) y a un ciclo de sonicación (tres minutos), de acuerdo con los protocolos estandarizados y reportados previamente ^22^. La suspensión obtenida del tubo endotraqueal se cultivó en medio de gérmenes comunes para la recuperación de bacterias ^24^ y en agar Sabouraud (BioMérieux, Marcy-l’Étoile, Francia) para la identificación de hongos. Las cepas se incubaron a 25 °C entre 3 y 30 días.

Los microorganismos aislados fueron sometidos a identificación y sensibilidad antifúngica, utilizando el sistema automatizado Vitek® 2 (BioMérieux, Marcy-l’Étoile, Francia). Los antifúngicos evaluados, fluconazol, voriconazol, caspofungina, micafungina y anfotericina B, estaban contenidos en las tarjetas del sistema Vitek®2.

### Recolección de la información y análisis estadístico

A partir de la información recolectada, se construyó una base de datos en Excel® (Microsoft Office 2010). Se establecieron las variables: microorganismo aislado, carga microbiana y resistencia a antifúngicos, y se hizo el análisis con el paquete estadístico R®, versión 4.1.3. Se usó estadística descriptiva para caracterizar los aislamientos mediante frecuencias absolutas y relativas.

## Resultados

**Figura 1:**
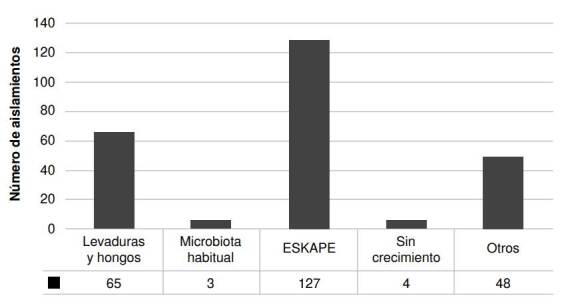
Clasificación de los microorganismos identificados mediante el sistema automatizado Vitek® 2 (BioMérieux, Marcy l'Étoile, Francia)

Se analizaron 121 tubos endotraqueales, de 113 pacientes de los dos centros hospitalarios. Se identificaron 247 aislamientos, de los cuales 127 eran bacterias (50,55 %) del grupo ESKAPE (*Enterococcus* spp., *S. aureus*, *K. pneumoniae*, *Acinetobacter baumannii*, *P. aeruginosa* y *Enterobacter* spp.), y 65 (26,32 %) eran hongos o levaduras (figura 1). Los resultados de los cultivos bacterianos fueron previamente publicados por el grupo de investigación ^23^.

Cincuenta tubos endotraqueales (41,3 %) fueron colonizados por hongos, de los cuales se aislaron 65 hongos diferentes (filamentosos y levaduras). De estos, el 64,62 % (n=42) correspondió a *C.albicans*; 30,77 % (n=20) a *C.* no-*albicans*; 3,08 % (n=2) a *Cryptococcus*, y 1,54 % (n=1) a mohos (cuadro 1).

**Cuadro 1:** Aislamientos de hongos a partir de muestras tomadas de tubos endotraqueales, identificados con el sistema automatizado Vitek® 2 (BioMérieux, Marcy l'Étoile, Francia)

Aislamientos		n	%
*Candida albicans*		42	64,61
*Candida no-albicans*		20	30,77
	*Candida glabrata*	11	55,00
	*Candida tropicalis*	7	35,00
	*Candida parapsilopsis*	1	5,00
	*Candida krusei*	1	5,00
*Cryptococcuss*		2	1,65
	*Cryptococcus neoformans*	1	50,00
	*Cryptococcus laurenti*	1	50,00
Mohos		1	0,83
	*Aspergilus fumigatus*	1	100,00
Más de un microorganismo		13	26,00
Dos géneros diferentes		1	7,69
Dos especies de *Candida* diferentes		12	92,31
	*Candida albicans* y *Candida glabrata*	7	53,85
	*Candida albicans* y *Candida tropicalis*	4	30,77
	*Candida tropicalis* y *Candida krusey*	1	7,69
	*Candida glabrata* y *Aspergillus*	1	7,69
Total		65	100,00

De las *C*. no-*albicans*, se encontró *C. glabrata* (55,00 %; n=11), *C. tropicalis* (35,00 %; n=7), *C. parapsilosis* (5,00 %; n=1) y *C. krusei* (5,00 %; n=1). Dentro del género *Cryptococcus*, se identificó un aislamiento de *C. neoformans* y otro de *C. laurentii*; y entre los mohos, uno de *Aspergillus fumigatus* (cuadro 1).

Se encontraron 13 (26 %) tubos endotraqueales colonizados por más de una especie fúngica. En una de las muestras, se identificó la colonización de géneros diferentes: *C. glabrata* y *A. fumigatus*. En cuanto a la colonización por diferentes especies de *Candida*, la combinación más frecuente fue *C. albicans* y *C. glabrata* (53,85 %), seguida de *C. albicans* y *C. tropicalis* (30,77 %), y *C. tropicalis* y *C. krusei* (7,69 %) (cuadro 1).

Al comparar los asilamientos entre los centros hospitalarios, el 58 % (n=29) de los hongos fue aislado en la Clínica Colombia y el 42 % (n=21) en la Fundación Santa Fe de Bogotá, pero hubo mayor cantidad de aislamientos positivos sobre el total de la muestra en la Fundación Santa Fe (47,72 %) que en la Clínica Colombia (37,66 %).

En la unidad de cuidados intensivso de la Clínica Colombia, se observó que el 25 % de las levaduras estaba asociado a bacterias del grupo ESKAPE, siendo *K. pneumoniae* la más frecuente. Se encontraron cocultivos frecuentes de *C. albicans* con *C. tropicalis* o con *C. glabrata* en los cultivos analizados (cuadro 2a).

**Cuadro 2:** Asociaciones de levaduras con otros microorganismos y su distribución por centros hospitalarios

a. Asociaciones de levaduras con otros microorganismos en cultivo, aislados a partir de las muestras de la Clínica Colombia			
Aislamientos		n	%
ESKAPE			
	*E. faecalis*	3	13,04
	*P. aeruginosa*	3	13,04
	*E. coli*	2	8,70
	*K. pneumoniae*	5	21,74
Otras levaduras			
	*C. tropicalis*	2	8,70
	*C. glabrata*	2	8,70
	*C. neoformans*	1	4,35
	*C. kruseii*	1	4,35
Otras bacterias			
	*S. marcescens*	2	8,70
	*M. catarrhalis*	1	4,35
Filamentosos			4,35
	*Aspergillus* spp.	1	
Total		23	100,00
**b.** Asociaciones de levaduras con otros microorganismos en cultivo aislados a partir de las muestras de la Fundación Santa Fe de Bogotá			
Aislamientos		n	%
ESKAPE			
	*K. pneumoniae*	2	16,66
	*P. aeruginosa*	2	16,66
Otras levaduras			
	*C. tropicalis*	2	16,66
	*C. glabrata*	6	50,00
Total		12	100,00

ESKAPE: microorganismos patógenos, virulentos y resistentes a antibióticos, que pertenecen a alguno de los siguientes géneros y especies: *Enterococcus* spp., *Staphylococcus aureus*, *Klebsiella pneumoniae, Acinetobacter baumannii*, *Pseudomonas aeruginosa* y *Enterobacter* spp.

En las muestras de la unidad de cuidados intensivos de la Fundación Santa Fe de Bogotá, se encontró una baja asociación en el cultivo, entre levaduras con bacterias del grupo ESKAPE. Se identificaron únicamente dos casos de cocultivo de *C. albicans* y *K. pneumoniae* y de *C. albicans* y *P. aeruginosa*, y un caso de *C. glabrata* y *K. pneumoniae*. La mayoría de los aislamientos fueron de una única levadura (40,91 %), seguidos por aquellos de más de una (31,82 %). La asociación más común fue la de *C. albicans* y *C. glabrata*. Estos hallazgos sugieren la necesidad de seguir explorando la dinámica microbiana en las unidades de cuidado intensivo y la potencial importancia clínica de las asociaciones entre levaduras y bacterias (cuadro 2b).

Se evaluó la sensibilidad antifúngica en 60 de los 65 hongos aislados. No se determinó el perfil de sensibilidad para *A. fumigatus*, debido a que no hay uno establecido para hongos filamentosos; y tampoco el de *C. laurentii*, porque el fabricante no dispone de la tarjeta de sensibilidad específica para esta especie fúngica (cuadro 3).

**Cuadro 3:** Especies de hongos identificadas y su perfil de sensibilidad a los compuestos antifúngicos

Especies	Azoles								Equinocandinas								Polienos				Total
	Perfil de resistencia a fluconazol (n=62)				Perfil de resistencia a voriconazol (n=61)				Perfil de resistencia a caspofungina (n=60)				Perfil de resistencia a micafungina (n=59)				Perfil de resistencia a anfotericina B (n=60				
	S		R		S		R		S		R		S		R		S		R		
	n	%	n	%	n	%	n	%	n	%	n	%	n	%	n	%	n	%	n	%	
*Candida albicans*	41	100	0	0	41	100	0	0	41	100	0	0	41	100	0	0	39	98	1	2,5	41
*Candida no-albicans*	18	90	2	20	19	5	1	20	20	100	0	0	19	100	0	0	19	100	0	0	20
*Candida glabrata*	11	100	0	0	11	100	0	0	11	100	0	0	11	100	0	0	11	100	0	0	11
*Candida tropicalis*	6	86	1	14	6	85	1	14	7	100	0	0	6	100	0	0	0	100	0	0	7
*Candida parapsilopsis*	1	100	0	0	1	100	0	0	1	100	0	0	1	100	0	0	1	100	0	0	1
*Candida krusei*	0	0	1	100	1	100	0	0	1	100	0	0	1	100	0	0	1	100	0	0	1
*Cryptococcus neoformans*	1	100	0	0	ND	ND	ND	ND	ND	ND	ND	ND	ND	ND	ND	ND	1	100	0	0	1
																					62

T: total; S: sensible; R: resistente; ND: no determinado

Se encontró que el 92,30 % de los hongos aislados fue sensible, al menos, a un fármaco, mientras que la eficacia media de todos los antifúngicos evaluados (fluconazol, voriconazol, caspofungina, micafungina, anfotericina B) fue de 99,26 %. Se destaca que todos los aislamientos analizados de *Candida* (*albicans*, *glabrata* y *parapsilosis*) fueron sensibles a fluconazol (cuadro 3). Solo un aislamiento de *C. tropicalis*, de los siete obtenidos por cultivo, presentó resistencia a azoles (fluconazol y voriconazol), pero fue sensible a equinocandinas (caspofungina, micafungina) y polienos (anfotericina B). Se aisló una cepa de *C. krusei* que presentó sensibilidad a todos los antifúngicos, con excepción de fluconazol. Se ha reportado que esta especie tiene resistencia intrínseca. Por su parte, *C. neoformans* fue sensible a fluconazol y a la anfotericina B (cuadro 3).

En la evaluación de todas las cepas, se obtuvo que: la concentración inhibitoria mínima para fluconazol fue menor o igual a 1 μg/ml (p25 < 1; p75 > 4); para voriconazol, fue menor o igual a 0,12 μg/ml; para caspofungina, fue menor o igual a 0,25 μg/ml; para micafungina, fue menor o igual a 0,06 μg/ ml; y para anfotericina B, fue menor o igual a 0,25 μg/ml (p25 < 0,25; p75 = 0,50). Para la cepa *C. tropicalis* resistente a azoles, no se pudo determinar la concentración inhibitoria mínima para micafungina y anfotericina B. La mayor eficacia la obtuvo el grupo de las equinocandinas (caspofungina y micafungina), pero sin diferencias observadas con respecto a los otros grupos farmacológicos: polienos (anfotericina B) y azoles (fluconazol y voriconazol) (cuadro 3).

## Discusión

Los microorganismos de la vía aérea son un grave problema para los pacientes hospitalizados en la unidad de cuidados intensivos y, particularmente, para aquellos que requieren asistencia respiratoria mecánica. Esto se debe a la colonización y la formación de biopelículas sobre los tubos endotraqueales, que funcionan como reservorio de los microorganismos. La neumonía asociada con el respirador es una de las principales infecciones asociadas con la atención en salud en dichas unidades. La Organización Mundial de la Salud indicó que la incidencia de esta infección en países en desarrollo es de 14,7 por 1.000 días de uso de un respirador ^2^^,^^25^.

En este estudio, el análisis de la micobiota de las vías respiratorias de pacientes en estado crítico, que requirieron intubación y asistencia respiratoria mecánica, mostró poca diversidad fúngica, a diferencia de la encontrada en los cultivos bacterianos de las mismas muestras ^24^. Como se indica en la figura 1, el porcentaje de levaduras obtenido sobre el total de los aislamientos (26 %), fue menor que el de bacterias (56 %), excluyendo las que se consideran parte de la microbiota habitual.

En general, los resultados sugieren que la micobiota de este grupo de pacientes está compuesta, en su mayoría, por levaduras, principalmente, *C. albicans*. Al relacionar las comunidades bacterianas y fúngicas en los mismos pacientes (cuadro 2), se encontraron resultados similares a lo reportado en publicaciones previas, donde la colonización de bacterias como *P. aeruginosa*, *K. pneumoniae* y *S. aureus* es facilitada por la presencia de *Candida* spp. ^26^^,^^27^. Algunos estudios reportan una mayor probabilidad de neumonía asociada con el respirador, de origen bacteriano, por coexistencia con hongos, ya que estas comunidades microbianas favorecen los procesos metabólicos y la comunicación intercelular ^28^.

Respecto a los aislamientos de hongos únicos de los tubos endotraqueales obtenidos de pacientes en la unidad de cuidados intensivos, se encontró una gran frecuencia de colonización por levaduras. En primer lugar, estuvo *C. albicans*, seguida de otras *C*. no-*albicans* y *Cryptococcus* spp., lo cual coincide con otros estudios de micobiota, en los que se reporta el género *Candida* como uno de los colonizadores comunes de las vías respiratorias ^20^. Las especies de *Candida* que se han aislado con mayor frecuencia de los tubos endotraqueales son: *C. albicans*, *C. glabrata* y *C. parapsilosis*^29^. En este trabajo, se observó con mayor frecuencia *C. albicans* y *C. glabrata*, incluso en coinfección.

Los hongos reconocidos como principales oportunistas incluyen especies de *Candida*, *Aspergillus* y *Fusarium*, relacionadas con altas tasas de mortalidad en infecciones asociadas con la atención en salud. Los dispositivos médicos son frecuentemente colonizados por hongos ^22^, principalmente por *Candida* spp. Esta especie se aísla frecuentemente de pacientes críticamente enfermos con sospecha de neumonía asociada con el respirador, inflamación persistente e inmunosupresión ^30^. Esto coincide con lo reportado en el presente estudio.

Se ha sugerido que *Candida* spp. puede ser un agente causal de neumonía asociada con el respirador ^3^^,^^29^. Sin embargo, no ha sido posible determinar si la presencia de esta levadura se debe solo a su colonización de las vías respiratorias. En diversos estudios se ha logrado aislar diferentes especies de *Candida* spp. en biopelículas de tubos endotraqueales con resistencia a fluconazol ^24^. Parte de la capacidad patógena de las especies de *Candida* se ha atribuido a su capacidad de formar biopelículas. Estas levaduras pueden generar infecciones persistentes y recurrentes relacionadas con el uso de dispositivos médicos. La asociación entre *Candida* spp. y el incremento de la mortalidad en pacientes con neumonía asociada con el respirador, genera estancias hospitalarias prolongadas y, por lo tanto, un aumento de los costos de atención en salud ^31^.

Se ha descrito que los pacientes críticamente enfermos colonizados por *Candida* spp. tienen más días de asistencia respiratoria mecánica y mayor mortalidad frente a los que no han sido colonizados por dicho microorganismo ^32^. Este es un criterio importante que podría tener en cuenta el personal médico, aunque es discutible atribuir la mortalidad de dichos pacientes a la colonización por *Candida* spp. Existen muchos factores que confunden y desorientan el diagnóstico y el seguimiento, según la condición específica de cada paciente.

En este estudio, se encontraron dos especies del género *Cryptococcus*, *C. neoformans* y *C. laurentii*. El hongo levaduriforme *Cryptococcus* spp. se encuentra distribuido a nivel mundial y se considera cosmopolita ^33^. Este hongo está relacionado con la enfermedad fúngica invasiva conocida como criptococosis, específicamente la especie *C. neoformans* (variedades *neoformans* y *gatti*), aunque otras especies como *C. laurentii* y *C. albidus* también se han reportado como inductoras de enfermedad en humanos, principalmente en aquellos inmunosuprimidos ^33^^,^^34^.

Para *C. neoformans* se han descrito factores de riesgo asociados con su presencia, como: infección por HIV, tratamiento con esteroides, trasplante de órgano sólido, neoplasias hematológicas, lupus eritematoso sistémico, artritis reumatoide, sarcoidosis, tratamiento con anticuerpos monoclonales -etanercept, infliximab, alemtuzumab-, diabetes mellitus, falla renal y enfermedad hepática crónica ^34^. Estos antecedentes podrían ser relevantes en pacientes como los incluidos en el presente estudio, ya que tienen compromiso inmunitario derivado de los tratamientos a los que pudieron haber estado sometidos.

Las infecciones asociadas con *C. laurentii* son características de los pacientes críticamente enfermos, con hospitalizaciones prolongadas, uso de dispositivos médicos invasivos, nutrición parenteral, exposición a esteroides e ingreso en la unidad de cuidados intensivos con necesidad crónica de esteroides ^21^^,^^35^. En Colombia, hay un reporte de infección por este hongo que podría servir de alerta en el manejo de este tipo de pacientes con factores de riesgo asociados ^21^.

El género *Aspergillus* se ha reportado como agente causal de infecciones respiratorias, neumonías y alergias ^36^. Sin embargo, en el presente estudio, solo se encontró un hongo filamentoso: *A. fumigatus*. Cabe resaltar que los tubos endotraqueales analizados no necesariamente provenían de pacientes con enfermedad respiratoria. Por lo tanto, es posible que este microorganismo haga parte de la micobiota de los pacientes sanos, lo cual es congruente con lo publicado por otros autores ^19^^,^^21^. Investigadores franceses caracterizaron la micobiota de los pulmones y hallaron *Aspergillus* spp. en personas sanas ^37^. En estudios recientes con pacientes con COVID-19 y asistencia respiratoria prolongada, se reportó aspergilosis ocasional ^21^^,^^38^.

Durante la pandemia por SARS-CoV2, se presentó un incremento en los reportes de aspergilosis pulmonar relacionada con COVID-19. Los factores de riesgo para el desarrollo de la aspergilosis pulmonar incluyen: daño pulmonar grave relacionado con COVID-19, uso de esteroides en síndrome de disnea aguda y administración indiscriminada de antimicrobianos de amplio espectro en la unidad de cuidados intensivos ^38^^-^^44^.

En la literatura científica actual, se reporta de manera constante una mayor mortalidad de los pacientes con aspergilosis pulmonar asociada con COVID-19, en comparación con aquellos sin aspergilosis ^45^. Aunque los datos del presente análisis se obtuvieron en fechas previas a la pandemia del 2020, es importante mencionar que ya se encontraba *A. fumigatus* en muestras de pacientes intubados en la unidad de cuidados intensivos. Los pacientes hospitalizados en dicha unidad luego de la infección por COVID-19, por lo general, eran de edad avanzada, con una amplia carga de morbilidad y con mayor riesgo de infecciones bacterianas y fúngicas dada sus características inmunológicas. En estos pacientes, las infecciones fúngicas se relacionaron con mayor mortalidad ^46^.

El conocimiento de la micobiota de las vías respiratorias es importante para prevenir potenciales infecciones o coinfecciones en los pacientes intubados. Aproximadamente, el 15 % de los pacientes con tubo endotraqueal reciben antibióticos para las neumonías asociadas con el respirador o las traqueobronquitis que son diagnosticadas clínicamente ^47^. En otros casos, la terapia antibiótica se inicia al momento de la intubación, lo que favorece el crecimiento de los hongos que hacen parte de la micobiota del paciente. En este estudio, las levaduras aisladas fueron sensibles, al menos, a un fármaco y el 100 % de las cepas de *Candida* (*C. albicans*, *C. glabrata* y *C. krusei*) fueron sensibles a fluconazol.

Los datos producto del presente análisis son un punto de partida para estudios adicionales que busquen explorar las interacciones entre huésped, micobioma y microbioma, incluyendo las comorbilidades, los tratamientos específicos, los antimicrobianos y las complicaciones de los cambios (disbiosis) en las comunidades de la microbiota respiratoria que facilitan la infección. Esto podría respaldar la toma de decisiones clínicas respecto al uso de antimicrobianos antes de la intubación del paciente, y al seguimiento y la prevención de coinfecciones fúngicas y bacterianas relacionadas con el desarrollo de neumonía asociada con el respirador.

Este trabajo indica que existe colonización por levaduras y mohos en los tubos endotraqueales de los pacientes en la unidad de cuidados intensivos, con mayor prevalencia de las levaduras. En algunos casos, se observó colonización por más de una especie en el mismo tubo. Estos resultados sugieren que los mohos y las levaduras deben tenerse en cuenta como posibles agentes etiológicos de la neumonía asociada con el respirador; o que estas, en coinfección con bacterias patógenas, sean facilitadoras en el desarrollo de este tipo de neumonía. Por lo tanto, es necesario promover prácticas médicas para la prevención de la neumonía asociada con el respirador en las unidades de cuidados intensivos, como el retiro temprano del respirador, la higiene oral, la cabecera inclinada a 30-45 °, y el tratamiento preventivo en cultivos positivos para *Candida* spp., entre otros.

Es importante resaltar que los datos aquí mencionados corresponden solo a las cepas obtenidas en cultivo, cuya sensibilidad es limitada para microorganismos exigentes o de difícil crecimiento. Por lo tanto, se requieren estudios adicionales que incluyan la identificación de la micobiota por secuenciación del espaciador interno transcrito (*Internal transcribed spacer*, ITS) u otros marcadores de interés. Igualmente, se puede incluir otro tipo de muestras, como el hisopado orofaríngeo y el aspirado endotraqueal, para hacer un análisis más profundo de la micobiota de los pacientes intubados.

Es relevante realizar estudios adicionales orientados a determinar la necesidad de iniciar un tratamiento empírico en caso de que estos hongos fuesen los causantes de fungemia invasiva. De igual forma, es importante buscar correlación entre los microorganismos colonizadores y su probabilidad de generar enfermedades, como candidiasis invasiva. Esta breve descripción de la micobiota aislada de pacientes colombianos, puede contribuir al seguimiento de las infecciones (emergentes) asociadas con la asistencia respiratoria mecánica en las unidades de cuidados intensivos.

## References

[1] Instituto Nacional de Salud (2020). Infecciones asociadas a dispositivos en unidades de cuidado intensivo, Colombia.

[2] Asensio Martín MJ, Hernández Bernal M, Teruel SY, Minvielle A. (2018). Infecciones en el paciente crítico. Medicine.

[3] Percival SL, Williams DW, Randle J, Cooper T (2014). Biofilms in infection prevention and control.

[4] Danin PE, Girou E, Legrand P, Louis B, Fodil R, Christov C (2015). Description and microbiology of endotracheal tube biofilm in mechanically ventilated subjects. Respir Care.

[5] Augustyn B. (2007). Ventilator-associated pneumonia: Risk factors and prevention. Crit Care Nurse.

[6] Depuydt P, Myny D, Blot S. (2006). Nosocomial pneumonia: Aetiology, diagnosis, and treatment. Curr Opin Pulm Med.

[7] Heo S, Haase EM, Lesse AJ, Gill SR, Scannapieco FA. (2008). Genetic relationships between respiratory pathogens isolated from dental plaque and bronchoalveolar lavage fluid from patients in the intensive care unit undergoing mechanical ventilation. Clin Infect Dis.

[8] Sottile FD, Marrie TJ, Prough DS, Hobgood CD, Gower DJ, Webb LX (1986). Nosocomial pulmonary infection: Possible etiologic significance of bacterial adhesion to endotracheal tubes. Crit Care Med.

[9] Schnabel RM, Linssen CF, Guion N, van Mook WN, Bergmans DC. (2014). Candida pneumonia in intensive care unit?. Open Forum Infect Dis.

[10] El-Ebiary M, Torres A, Fábregas N, de la Bellacasa JP, González J, Ramírez J (1997). Significance of the isolation of Candida species from respiratory samples in critically ill, non-neutropenic patients: an immediate postmortem histologic study. Am J Respir Crit Care Med.

[11] Su K-C, Chou K-T, Hsiao Y-H, Tseng C-M, Su VY-F, Lee Y-C (2017). Measuring (1,3)-p-D- glucan in tracheal aspirate, bronchoalveolar lavage fluid, and serum for detection of suspected Candida pneumonia in immunocompromised and critically ill patients: A prospective observational study. BMC Infect Dis.

[12] Dermawan JKT, Ghosh S, Keating MK, Gopalakrishna KV, Mukhopadhyay S. (2018). Candida pneumonia with severe clinical course, recovery with antifungal therapy and unusual pathologic findings: A case report. Medicine (Baltimore).

[13] Haas CF, Eakin RM, Konkle MA, Blank R. (2014). Endotracheal tubes: Old and new. Respir Care.

[14] Sun F, Qu F, Ling Y, Mao P, Xia P, Chen H (2013). Biofilm-associated infections: Antibiotic resistance and novel therapeutic strategies. Future Microbiol.

[15] Gil-Perotin S, Ramírez P, Marti V, Sahuquillo JM, González E, Calleja I (2012). Implications of endotracheal tube biofilm in ventilator-associated pneumonia response: A state of concept. Crit Care.

[16] El-Sayed A, Aleya L, Kamel M. (2021). Microbiota’s role in health and diseases. Environ Sci Pollut Res.

[17] Del Campo-Moreno R, Alarcón-Cavero T, D-Auria G, Delgado-Palacio S, Ferrer-Martínez M. (2018). Microbiota en la salud humana: técnicas de caracterización y transferencia. Enferm Infecc Microbiol Clin.

[18] Restrepo-Rivera LM, Cardona-Castro N. (2021). Micobioma: diversidad fúngica en el cuerpo humano. CES Med.

[19] Bandara HMHN, Panduwawala CP, Samaranayake LP. (2019). Biodiversity of the human oral mycobiome in health and disease. Oral Dis.

[20] Belvoncikova P, Splichalova P, Videnska P, Gardlik R. (2022). The human mycobiome: Colonization, composition and the role in health and disease. J Fungi (Basel).

[21] Ruiz-Rodríguez A, Lusarreta-Parga P, de Steenhuijsen Piters WAA, Koppensteiner L, Balcazar-Lopez CE, Campbell R (2022). Bacterial and fungal communities in tracheal aspirates of intubated COVID-19 patients: A pilot study. Sci Rep.

[22] Vandecandelaere I, Matthijs N, van Nieuwerburgh F, Deforce D, Vosters P, De Bus L (2012). Assessment of microbial diversity in biofilms recovered from endotracheal tubes using culture dependent and independent approaches. PLoS ONE.

[23] Martin-Loeches I, Dickson R, Torres A, Hanberger H, Lipman J, Antonelli M (2020). The importance of airway and lung microbiome in the critically ill. Crit Care.

[24] Cifuentes EA, Sierra MA, Yepes AF, Baldión AM, Rojas JA, Álvarez-Moreno CA (2022). Endotracheal tube microbiome in hospitalized patients defined largely by hospital environment. Respir Res.

[25] Karakuzu Z, Iscimen R, Akalin H, Girgin NK, Kahveci F, Sinirtas M. (2018). Prognostic risk factors in ventilator-associated pneumonia. Med Sci Monit.

[26] Pérez-Rodríguez G, Dias S, Pérez-Pérez M, Fdez-Riverola F, Azevedo NF, Lourenço A. (2018). Agent-based model of diffusion of N-acyl homoserine lactones in a multicellular environment of Pseudomonas aeruginosa and Candida albicans. Biofouling.

[27] Meto A, Colombari B, Sala A, Pericolini E, Meto A, Peppoloni S (2019). Antimicrobial and antibiofilm efficacy of a copper/calcium hydroxide-based endodontic paste against Staphylococcus aureus, Pseudomonas aeruginosa and Candida albicans. Dent Mater J.

[28] Sedlmayer F, Hell D, Müller M, Auslander D, Fussenegger M. (2018). Designer cells programming quorum-sensing interference with microbes. Nat Commun.

[29] Baghdadi E, Khodavaisy S, Rezaie S, Abolghasem S, Kiasat N, Salehi Z (2016). Antifungal susceptibility patterns of candida species recovered from endotracheal tube in an intensive care unit. Adv Med.

[30] Albert M, Willamson D, Muscedere J, Lauzier F, Rostein C, Kanji S (2014). Candida in the respiratory tract secretions of critically ill patients and the impact of antifungal treatment: A randomized placebo-controlled pilot trial (CANTREAT study). Crit Care.

[31] Delisle M-S, Williamson DR, Albert M, Perreault MM, Jiang X, Day AG (2011). Impact of Candida species on clinical outcomes in patients with suspected ventilator-associated pneumonia. Can Respir J.

[32] Huang D, Qi M, Hu Y, Yu M, Liang Z. (2020). The impact of Candida spp. airway colonization on clinical outcomes in patients with ventilator-associated pneumonia: A systematic review and meta-analysis. Am J Infect Control.

[33] Góralska K, Blaszkowska J, Dzikowiec M. (2018). Neuroinfections caused by fungi. Infection.

[34] Sánchez EG, Acosta D, Álvarez J, Sánchez G, García-Casallas J. (2022). Criptococosis diseminada por terapia biológica, se debe gestionar el riesgo. Biomedica.

[35] Burbano S, Gomez N, Álvarez P, González A, Turmino C, Asquineyer Y. (2020). Compromiso pulmonar por Cryptoccus laurentii en paciente inmunocomprometido. Rev Am Med Respir.

[36] Vandewoude KH, Blot SI, Depuydt P, Benoit D, Temmerman W, Colardyn F (2006). Clinical relevance of Aspergillus isolation from respiratory tract samples in critically ill patients. Crit Care.

[37] El-Jurdi N, Ghannoum MA. (2017). The Mycobiome: Impact on health and disease states. Microbiol Spectr.

[38] Lansbury L, Lim B, Baskaran V, Lim WS. (2020). Co-infections in people with COVID-19: A systematic review and meta-analysis. J Infect.

[39] Cox MJ, Loman N, Bogaert D, O’Grady J. (2020). Co-infections: potentially lethal and unexplored in COVID-19. Lancet Microbe.

[40] Zhu X, Ge Y, Wu T, Zhao K, Chen Y, Wu B (2020). Co-infection with respiratory pathogens among COVID-2019 cases. Virus Res.

[41] Zhang G, Hu C, Luo L, Fang F, Chen Y, Li J (2020). Clinical features and short-term outcomes of 221 patients with COVID-19 in Wuhan, China. J Clin Virol.

[42] Koehler P, Cornely OA, Bottiger BW, Dusse F, Eichenauer DA, Fuchs F (2020). COVID-19 associated pulmonary aspergillosis. Mycoses.

[43] Alanio A, Dellière S, Fodil S, Bretagne S, Mégarbane B. (2020). Prevalence of putative invasive pulmonary aspergillosis in critically ill patients with COVID-19. Lancet Respir Med.

[44] Rutsaert L, Steinfort N, van Hunsel T, Bomans P, Naesens R, Mertes H (2020). COVID-19- associated invasive pulmonary aspergillosis. Ann Intensive Care.

[45] Janssen NAF, Nyga R, Vanderbeke L, Jacobs C, Ergün M, Buil JB (2021). Multinational Observational Cohort Study of COVID-19-Associated Pulmonary Aspergillosis. Emerg Infect Dis.

[46] Pemán J, Ruiz-Gaitán A, García-Vidal C, Salavert M, Ramírez P, Puchades F (2020). Fungal co-infection in COVID-19 patients: Should we be concerned?. Rev Iberoam Micol.

[47] Kelly BJ, Imai I, Bittinger K, Laughlin A, Fuchs BD, Bushman FD (2016). Composition and dynamics of the respiratory tract microbiome in intubated patients. Microbiome.

